# Cost-utility analysis of extensile lateral approach versus sinus tarsi approach in Sanders type II/III calcaneus fractures

**DOI:** 10.1186/s13018-020-01963-5

**Published:** 2020-09-18

**Authors:** Zihua Li, Xinbo Wu, Haichao Zhou, Shaochen Xu, Fajiao Xiao, Hui Huang, Yunfeng Yang

**Affiliations:** Department of Orthopedics, Shanghai Tongji Hospital, Tongji University School of Medicine, Shanghai, 200065 China

**Keywords:** Cost-utility analysis, Calcaneus fracture, Extensile lateral approach, Sinus tarsi approach

## Abstract

**Background:**

Extensile lateral approach had been recognized as the gold standard technique for displaced intra-articular calcaneus fractures (DIACFs) while sinus tarsi approach had been increasingly valued by surgeons and comparative clinical outcome was shown in both techniques. Appropriate decisions could be made by the clinicians with the help of cost-utility analysis (CUA) about optimal healthcare for type II/III calcaneus fracture.

**Method:**

A single-center, retrospective study was conducted in which basic characteristics, clinical outcomes, and health care costs of 109 patients had been obtained and analyzed. Changes in health-related quality of life (HRQoL) scores, validated by EuroQol five-dimensional-three levels (EQ-5D-3L), were used to enumerate quality-adjusted life years (QALYs). Cost-effectiveness was determined by the incremental cost per QALY.

**Results:**

One hundred nine patients were enrolled in our study including 62 in the ELA group and 47 in the STA group. There were no significant differences between these two groups in mean total cost, laboratory, and radiographic evaluation expense, surgery, anesthesia, and antibiotic expense. The expense of internal fixation materials ($3289.0 ± 543.9) versus ($2630.6 ± 763.7) and analgesia ($145.8 ± 85.6) versus ($102.9 ± 62.7) in ELA group were significantly higher than in the STA group (*P* < .001, *P* = .008, respectively). Visual Analogue Scale (VAS) scores showed significant difference at postoperative 3 and 5 days (*P* < .001). American Orthopaedic Foot and Ankle Society (AOFAS) ankle-hindfoot scores and the Bohlers’ and Gissane angle showed no significant differences between the two groups before and after the operation. The cost-effectiveness ratios of ELA and STA were $8766.8 ± 2835.2/QALY and $7914.9 ± 1822.0/QALY respectively, and incremental cost-effectiveness ratio (ICERs) of ELA over STA was $32110.00/QALY, but both showed no significant difference.

**Conclusion:**

Both ELA and STA techniques are effective operative procedures for the patients with calcaneus fracture. Moreover, STA seems to be more reasonable for its merits including less postoperative pain, and less expense of analgesia as well as internal fixation materials.

**Level of evidence:**

5

## Background

Calcaneus fracture is the most commonly happened one in tarsal bone, accounting for 2% of all fractures [1–3] and 75% of which are displaced intra-articular calcaneus fractures (DIACFs) [4, 5]. According to the classification system introduced by Sanders in 1993, calcaneus fracture is classified into four types based on the number and location of articular fragments seen on coronal and axial computed tomography (CT) [6, 7]. Nonoperative and operative treatments are the two traditional managements of calcaneus fractures. However, patients with conservative treatment may develop posttraumatic arthritis, chronic heel deformity, and malalignment of mechanical axis of the limb, which may seriously affect the quality of life [8, 9]. Surgical treatment is therefore necessary, and open reduction and internal fixation (ORIF) via extensile lateral approach (ELA) have been considered as the gold standard in the management of type II/III calcaneus fracture for its clear visualized for reduction and favorable outcomes in long-term [10–13]. However, some articles report multiple complications of ELA including hematoma, wound slough, dehiscence, and infection, and the occurrence rate is approaching 33% [14–18]. Many minimally invasive approaches including closed reduction, percutaneous screws fixation, and modified lateral approach (minimal invasive sinus tarsi approach) have been introduced in order to reduce the incidence of surgical complications [19–23]. Many investigators and surgeons have advocated using sinus tarsi approach (STA) for its lower reported wound complications rate, lower visual analog pain scale levels, comparative clinical outcomes, and increased overall satisfaction [24–26], and there has been a trend toward this technique in recent times. However, the optimal surgical approach for accurate reduction of DIACFs still remains controversial.

Owing to persistent pain, stiffness, and gait abnormalities, DIACFs may result in permanent disability and reduction of quality of life [5, 27–29]. Additionally, DIACFs are more frequently happened in the most economically active population such as adults and adolescents, which undoubtedly lay high socio-economic costs for its difficulties to return to work [30, 31]. Therefore, with growing pressure to reduce costs while high-quality care is still delivered, cost-effective research in calcaneus fracture is becoming increasingly important to discuss, which may help clinicians make appropriate surgical strategies and reach optimal health care [32].

Cost-utility analysis (CUA), which belongs to effectiveness analysis, evaluates the outcome in units of utilities standing for quantity and quality of life [33, 34]. Alterations in health-related quality of life (HRQoL) scores validated by EuroQol five-dimension three-levels (EQ-5D-3L) were applied to enumerate quality-adjusted life years (QALYs), which present the result of CUA. In addition, incorporated with cost information, an incremental cost-effectiveness ratio (ICER) can be used to enumerate between two techniques and display a cost per QALY value: lower figure represents more cost-effective strategy [35, 36].

To the best of our knowledge, there had not been any cost-effectiveness research comparing the two approaches of calcaneus fractures conducted in China. Therefore, the purpose of this study was to assess the cost and cost-effectiveness of two techniques for treatment of Sanders type II/III in calcaneus fractures, and thus, it may help us make optimal clinical decisions.

## Method

### General information

We conducted a retrospective review of 109 patients (62 in the ELA group and 47 in the STA group) who had been diagnosed as type II/III calcaneus fracture and had undergone ORIF surgery via ELA or STA from January 2013 to October 2018. Basic demographic and clinical characteristic of the patients were obtained including age, gender, type of fracture, anesthesia, the length of hospital stay, and follow-up time.

### Inclusion and exclusion criteria

Participants were enrolled if they met the following eligibility criteria: (1) DIACFs classified as Sanders type II/III, (2) closed and fresh fractures, (3) fractures that underwent procedures via ELA or STA, and (4) follow-up period greater than 1 year. The exclusion criteria were as follows: (1) fractures classified as Sanders I or IV; (2) malunion, nonunion, open, and bilateral fractures; (3) associated or multiple trauma; and (4) severe systemic diseases and commodity.

### Clinical assessment

The pain was measured using Visual Analogue Scale (VAS) with the score ranged from 0 to 10 points indicating no pain to worst pain. The VAS scores were collected at pre-operation and postoperative 3 days, 5 days, and 7 days. Functional outcome was measured by American Orthopaedic Foot and Ankle Society (AOFAS) ankle-hindfoot scores at pre-operation, at postoperative 3 months, and at final follow-up with the scores ranging from 0 to 100 points of pain, function, and alignment. Radiographic including Bohler’s angle and Gissane angle was measured on fluoroscopic images at pre-operation, at postoperative 3 months, and at final follow-up time. The specific radiographic views including the anteroposterior (AP), lateral, axial projections, and CT scans were collected preoperatively. Based on the anatomic characteristic of calcaneus fractures shown in preoperative radiographs and CT, surgeons could attain the information of the type, level, locations of the fractures and measure height, width, and Bohler’s and Gissane angle of the calcaneus.

### Cost and cost-utility analysis

**.** The medical and financial records of patients including the expense of total costs, laboratory and radiographic evaluation, surgery, anesthesia, analgesia, internal fixation materials, and antibiotic were obtained from our hospital’s medical and financial information center. However, indirect costs including miss time from work or decreased productivity, rehabilitation, further consultation, and transportation were not enumerated because each patients’ situation and the employment status varied. All costs were converted into US dollars ($) at their value during January 2020.

Clinical outcome was monitored by recording health-related quality of life (HRQoL) scores before and after operation with a score ranging from − 0.11 to 1.0, and 1.0 indicates full health. Validation of HRQoL scores were converted by EQ-5D-3L including five dimensions: mobility, self-care, activities of daily life, pain, and anxiety/depression and each depicted by three levels from no, mild, or moderate to severe problems. Thus, this depictive system includes 243 combinations, or health states. Combining HRQoL index and time were used to estimate QALYs, which are enumerated for the area under the curve by trapezoidal method.

The CUA was conducted from the healthcare perspective and was presented by the cost-effectiveness ratio (CER) and incremental cost-utility ratio (ICER). An annual discount of 3% was adjusted to QALYs and mean total costs.

$$ \mathrm{ICER}=\frac{{\mathrm{Cost}}_{\mathrm{ELA}}-{\mathrm{Cost}}_{\mathrm{STA}}}{{\mathrm{QALY}}_{\mathrm{ELA}}-{\mathrm{QALY}}_{\mathrm{STA}}} $$

Different discount rates (0% and 5%) were used to mean total costs and QALYs for sensitivity analysis.

### Surgical technique

The decisions including the surgical procedures, type of the incision—ELA or STA, and type of the implants were made by senior surgeons based on the anatomic characteristic of the fractures and their preference. There were three experienced surgeons included in total and were coded as A, B, and C in this study. They all learned from the same professor and trained in the same place, and therefore, the surgeons can perform the procedures with equivalent effectiveness and low complication rate.

### Extensile lateral approach

**Fig.1:**
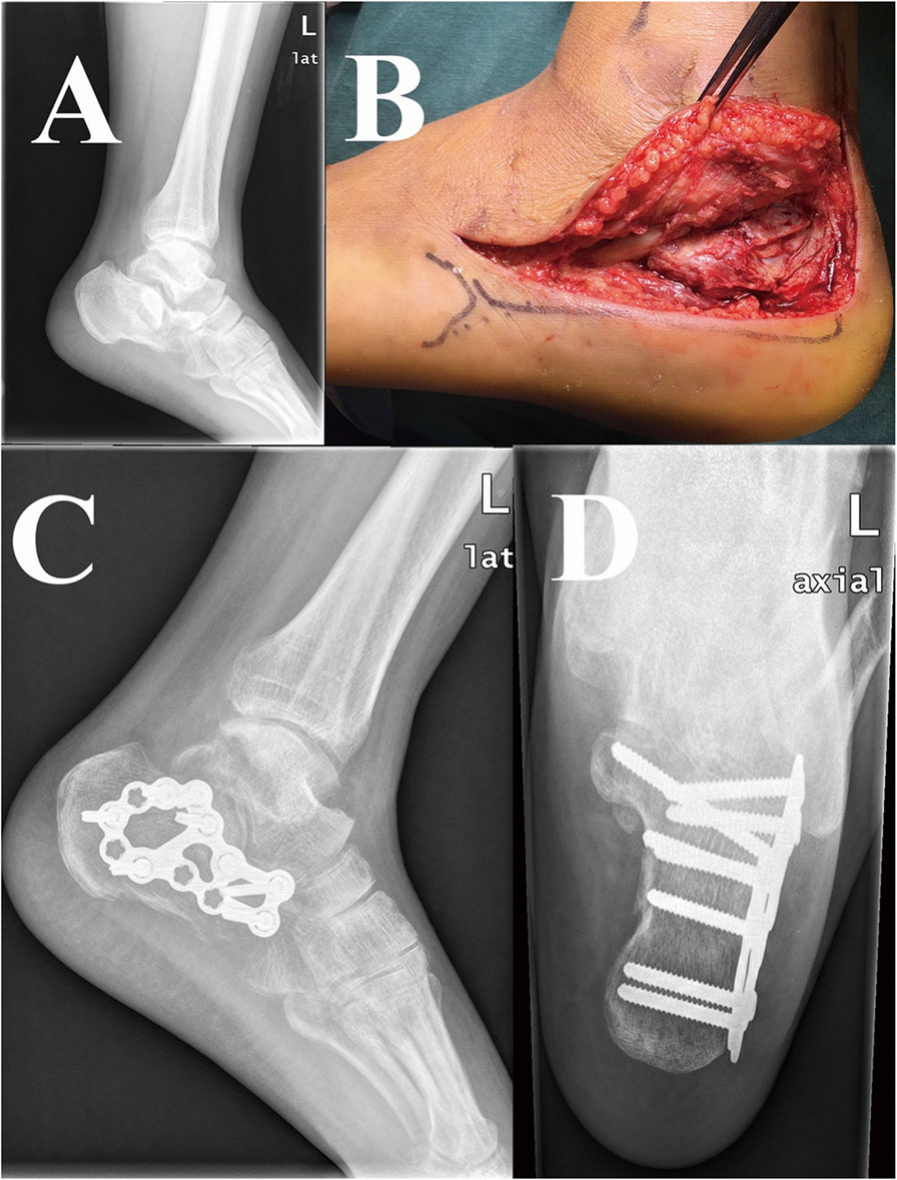
**a** The preoperative lateral image demonstrates collapse of the Bohler’s angle and posterior facet. **b** Intraoperative gross image of surgery via the extensile lateral approach (ELA). **c** The postoperative lateral image shows restoration of the Bohler’s angle and posterior facet by plate and screws. **d** The axial image shows restoration of the width and alignment of calcaneus

Patients with a tourniquet at the thigh were under epidural, local, general, or subarachnoid block anesthesia in the lateral position. The incision of ELA, which originated perpendicularly from 5 cm over lateral malleolus or the midpoint between the fibula and Achilles tendon and stopped at the base of 5th metatarsal, was performed by experienced surgeons followed the way which was depicted by Benirshcke and Sangeorzan. With the flap held by several 2.0-mm Kirschner wires, this incision allowed the visualization of the lateral wall of calcaneus and subtalar joint and anatomic reduction was achieved directly under the guidance of C-arm fluoroscopy. Consequently, the length, height, and width of the calcaneus were restored (Fig. 1).

### Sinus tarsi approach

**Fig. 2:**
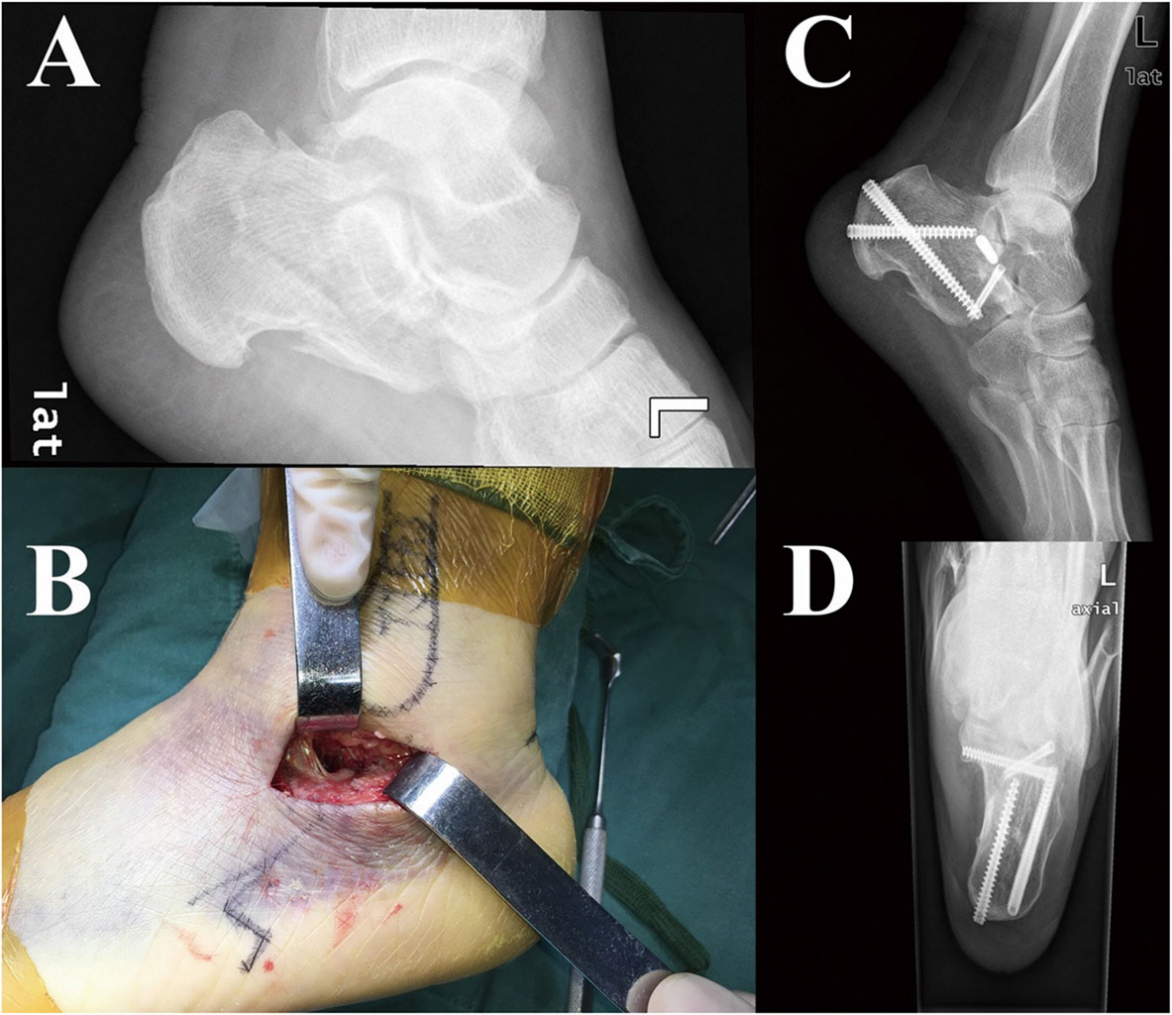
**a** The preoperative lateral image shows fracture line involving the subtalar joint. **b** Intraoperative gross image of surgery via the sinus tarsi approach (STA). **c** The postoperative lateral image shows restoration of Bohler’s angle, and fracture line involving subtalar joint was reduced by screws. **d** The axial image shows the normal alignment of calcaneus

Patients with a tourniquet at the thigh were under epidural, local, general, or subarachnoid block anesthesia in the lateral position. The incision was performed along a line from the tip of the lateral malleolus to the base of the fourth metatarsal, and its length was approximately 3–5 cm. This incision allowed the visualization of the posterior facet, anterior process, and even the calcaneocuboid joint with the calcaneofibular ligament dissected and the extensor brevis muscle elevated. Two 2.5-mm Schanz screw were inserted percutaneously into the calcaneal tuberosity and talus from medial to lateral, and tibia distraction device (Johnson & Johnson, USA) was applied to correct the deformity and restore the length, height, and width of the calcaneus with the guidance of C-arm fluoroscopy (Fig. 2).

### Statistical analysis

The Kolmogorov-Smirnov test was applied to examine the normality of all variables. The between-group differences were compared by Student’s *t* test for normal distribution variables (mean ± standard deviation) or a Mann-Whitney *U* test for abnormal distribution variables (median (25% quartile–75% quartile)) or a chi-square test for categorical variables (*n* (%)). The differences in the longitudinal changes of the Bohler’s, Gissane angle and VAS scores between ELA and STA group were compared by applying non-parametric test. All analyses were performed using SPSS 20.0 software (SPSS, IL, USA). Differences were considered to be statistically significant when *P* value was less than .05.

## Results

### Patient information

A total of 109 patients were enrolled in our research, including 62 in ELA group (45.40 ± 12.91 years old, 82.26% male) and 47 in STA group (49.92 ± 14.98 years old, 80.85% male). In the ELA group, 36 were classified as Sanders type II and 26 were Sanders type III whereas in the STA group 28 were classified as Sanders type II and 19 were Sanders type III. A sum of 52 in the ELA group underwent spinal anesthesia and 10 underwent general anesthesia whereas among the patients in the STA group, 40 underwent spinal anesthesia and 7 were under general anesthesia. The length of hospital stay and follow-up time were comparative between the ELA group and STA group. There were no significant differences in age, gender, fracture type, antibiotics, anesthesia, the length of hospital stay, and the follow-up time between these groups (Table 1).

**Table 1:** Patient information

Parameters	ELA (*N* = 62)	STA (*N* = 47)	*p* value
Age (years)	45.40 ± 12.91	49.92 ± 14.98	.095
Gender			.522
Male	51	38	
Female	11	9	
Fracture type			.516
Sanders II	36	28	
Sanders III	26	19	
Anesthesia			.860
Spinal anesthesia	52	40	
General anesthesia	10	7	
The length of hospital stay	10.9 ± 3.7	9.4 ± 3.6	.060
Follow-up time (month)	30.5 ± 9.7	28.4 ± 13.5	.150

Data are means ± SD and numbers of subjects. Significant difference (*P* < .05)

*ELA* Extensile lateral approach, *STA* Sinus tarsi approach

### Clinical outcome

All the clinical outcomes including VAS scores, AOFAS scores, the Bohler’s angle, and the Gissane angle are presented in Table 2. Results showed that VAS score was significantly different at postoperative 3 and 5 days between the two groups (*P* < .001, *P* = .002, respectively). However, there was no significant difference in VAS scores before operation and at the final follow-up time between these two groups (*P* = .151, *P* = .693, respectively). AOFAS scores improved greatly 3 months after surgery, from 50.0 ± 8.0 to 80.3 ± 6.3 in the ELA group and from 51.2 ± 7.9 to 81.4 ± 5.6 in the STA group, but there showed no significant difference between two groups at these time points (*P* = .413, *P* = .325, respectively). Similarly, the Bohler’s angle and the Gissane angle were significantly improved 3 months after surgery, from 13.6° ± 7.3 to 30.2° ± 7.6 (*P* < .001) and from 102.8° ± 14.2 to 130.0° ± 9.2 (*P* < .001) in the ELA group whereas in the STA group, the Bohler’s angle changed from 16.0° ±7.4 to 28.2° ± 7.1 (*P* < .001) and the Gissane angle changed from 102.1° ± 12.5 to 126.0° ± 7.5 (*P* < .001). There showed no significant difference in the Bohler’s and the Gissane angle before operation (*P* = .086, *P* = .0530) and at postoperative 3 months (*P* = .920, *P* = .255). Moreover, there were no significant differences among AOFAS scores, the Bohler’s angle, and the Gissane angle at final follow-up between these two groups (*P =* .113, *P =* .065, *P =* .139, respectively).

**Table 2:** Clinical outcome

Parameters	ELA (*N* = 62)	STA (*N* = 47)	*p* value
VAS scores			
Preoperative	6.9 ± 1.1	6.6 ± 1.0	.151
Postoperative 3 days	4.6 ± 0.9	3.0 ± 0.8	< .001
Postoperative 5 days	2.3 ± 0.7	1.8 ± 0.5	.002
Postoperative 7 days	1.6 ± 0.5	1.5 ± 0.5	.693
AOFAS			
Preoperative	50.0 ± 8.0	51.2 ± 7.9	.413
Postoperative 3 months	80.3 ± 6.3	81.4 ± 5.6	.325
Final follow-up	83.7 ± 4.6	85.0 ± 4.0	.113
Bohler’s angle (degrees)			
Preoperation	13.6 ± 7.3	16.0 ± 7.4	.086
Postoperative 3 months	30.2 ± 7.6	28.2 ± 7.1	.053
Final follow-up	29.9 ± 6.6	28.4 ± 6.3	.065
Gissane angle (degrees)			
Pre-operation	102.8 ± 14.2	102.0 ± 12.5	.920
Postoperative 3 months	130.0 ± 9.2	127.7 ± 8.2	.255
Final follow-up	126.0 ± 7.5	128.2 ± 7.8	.139
Preoperative EQ-5D score	0.63 (0.57–0.68)	0.59 (0.52–0.68)	.246
Postoperative EQ-5D	0.92 (0.89–0.95)	0.93 (0.88–0.95)	.755
Gained QALY	0.77 (0.73–0.80)	0.75 (0.69–0.79)	.250

*EQ-5D* EuroQol five-dimensional, *VAS* Visual Analogue Scale, *AOFAS* American Orthopaedic Foot and Ankle Society, *QALY* Quality-adjusted life-year

### Health care costs

Table 3 summarizes the itemized mean costs of ELA and STA groups. There were no significantly statistical differences between these two groups in the total costs and expense of laboratory and radiographic evaluation, surgery, antibiotics, and anesthesia. However, the expense of internal fixation materials and analgesia exists significant difference between the two groups (*P* < .001, *P* = .008, respectively).

**Table 3:** Health care cost

Parameters	ELA (*N* = 62)	STA (*N* = 47)	*p* value
Laboratory expense	$143.1 ± 42.9	$160.8 ± 109.5	.605
Radiography	$78.1 ± 21.8	$80.8 ± 15.1	.217
CT	$46.3 ± 21.8	$44.5 ± 21.7	.506
Surgery	$396.3 ± 130.5	$442.9 ± 202.7	.376
Anesthesia	$146.3 ± 67.5	$142.0 ± 70.1	.778
Analgesia	$145.8 ± 85.6	$102.9 ± 62.7	.008
Internal fixation materials	$3289.0 ± 543.9	$2630.6 ± 763.7	< .001
Antibiotic drugs	$85.0 ± 44.6	$76.5 ± 54.9	.104
Total cost	$6481.0 ± 1504.4	$5838.8 ± 1276.2	.200

Values represent the mean cost per patient in January 2020 US dollars. Significant difference (*P* < .05)

*CT* Computed tomography

### Cost-utility analysis

Changes in HRQoL scores validated by EQ-5D-3L in the two groups are presented as median (25% quartile, 75% quartile) in Table 2. The average baseline HRQoL scores of the ELA and STA group were similar (median 0.63; range, 0.57–0.68, and median 0.59; range, 0.52–0.68, respectively, *P =* .246). The average HRQoL scores at 1 year (0.92 in the ELA group and 0.93 in STA group) showed no significant difference (*P =* .755). The mean gained QALYs of the ELA and STA groups are 0.77 (range, 0.73–0.80) and 0.75 (range, 0.69–0.79), respectively. However, no significantly statistical difference exists in gained QALYs (*P =* .250).

The consequences of CUA are described on Table 4. The CERs of ELA and STA groups were $8766.8 ± 2835.2/QALY and $7914.9 ± 1822.0/QALY (*P =* .245), respectively. The difference between the mean total costs of the ELA and STA groups represents the incremental cost: $642.2 (ELA over STA). The mean gained QALYs and ICERs of ELA over STA were 0.02 and $32,110.00/QALY. With 3% and 5% annual discounts, the ICERs of ELA were $32,108.40/QALY and $32,126.05/QALY in the STA group, respectively (Table 5).

**Table 4:** Cost-effectiveness ratios (CERs)

Parameters	ELA (*N* = 62)	STA (*N* = 47)	*p* value
Cost	$6481.0 ± 1504.4	$5838.8 ± 1276.2	.200
Utility (QALY)	0.77 (0.73–0.80)	0.75 (0.69–0.79)	.755
CERs	$8766.8 ± 2835.2	$7914.9 ± 1822.0	.245

*QALY* Quality-adjusted life-year

**Table 5:** Incremental cost-effectiveness ratio (ICER)

Parameters	Normal	3% Discount	5% Discount
ΔCost	642.20	623.51	605.36
ΔQALY	0.02	1.942 × 10^−2^	1.94 × 10^−2^
ICER (Δcost/ΔQALY)	32,110.00	32,106.59	31,794.26

*ICER* Incremental cost-effectiveness ratio

## Discussion

Up to now, the optimal treatment of displaced intra-articular calcaneus fractures remains controversial even though the equipment and technologies have been developing rapidly. The ELA can provide good visualization of the fracture site, but several studies have reported that the postoperative wound complication rate including wound edge necrosis, dehiscence, hematoma, or infection is relatively high. The STA can minimize soft tissue damage and reduce the risk of postoperative complication while allowing comparative fracture reduction. Previous studies comparing the therapeutic efficacy and clinical outcomes of ORIF via ELA or STA have showed no significant differences [37–40]. From our results, the clinical outcomes including the Bohler’s and Gissane angle, VAS scores, and AOFAS scores were in accord with previous studies. Nevertheless, to the best of our knowledge, no studies comparing the direct costs and effectiveness of two techniques in China were conducted. Therefore, we have conducted this cost-utility analysis and provide another perspective for surgeons to make an optimally clinical decision in economic perspective.

In recent decades, surgery for treating calcaneus fractures has showed that it can bring great cost-effectiveness compared with nonoperative treatments. For both surgeons and patients, they have high requirements of satisfaction on clinical outcomes and cost-effectiveness due to the advancement of medicine, and therefore, costs are a crucial factor during making clinical decision. Cost-analysis is attached great significance of making clinical decision and is frequently used to evaluate which intervention can offer figure through comparing the cost and health impact of interventions [41]. Thus, surgeons are supposed to make full assessments of costs and take benefits into consideration when making clinical strategy to manage specific patients [42].

An article published in 2017 has used pooled data of western country to compare the cost and benefits in patients with calcaneus fractures classified as Sanders type II/III, which were managed with surgeries via extensile lateral approach or sinus tarsi approach or non-operative treatments [32]. It demonstrated that ORIF via STA is the least expensive option for treating Sanders type II/III concerning total costs, probability of working the same job, and duration out of work after ORIF. However, previous study did not conduct systematic retrospective review and report itemized details of the costs, which may hamper the ability to draw firm conclusions about cost-effectiveness by limited data. Moreover, long-term results after newer or refine ORIF techniques are unknown. In our study, we collected and checked the medical bills from single information center of all patients in order to conduct a cost-analysis.

In this retrospective view, only direct health costs were collected, and for the indirect health costs including miss time from work or decreased productivity, rehabilitation, further consultation, and transportation were not calculated. CUA can be performed even without indirect costs [43–45]. According to the results, we found no statistical differences in total costs, laboratory and radiographic evaluation expense, surgical expenses, antibiotic drugs expense, anesthesia expense, and the length of hospital stay. Our study found significant differences in the analgesia expenses and internal fixation materials costs. The costs of patients in ELA group were higher than the cost of patients in STA group ($145.8 ± 85.6 versus $102.9 ± 62.7, *P* = .080). Patients underwent surgical treatment via ELA which have caused much more severe injury to soft tissue and blood vessels comparing with STA. Owing to the larger wound caused by ELA, patients’ complaints of pain in the ELA group were more obvious. According to the pain measurement and VAS scores, there did exist significant difference in scores at 3 days and 5 days after surgery. Therefore, clinical strategies were made including using more effective analgesic drugs or extending the time of applying analgesic drugs, which resulted in the statistical difference in the expense of analgesia between these two groups. However, after postoperative treatments and caring, there showed no significant difference in VAS scores at 7 days after surgery.

Concerning the surgeon opinion for both techniques, surgeon A could master ELA and STA techniques proficiently while surgeon B prefers performing ELA technique and surgeon C prefers performing STA technique. Additionally, patients’ specific condition and subjective wishes are supposed to be taken into consideration, and therefore, there was no significant difference among surgeon rating for both surgical techniques to reconstruct the height, width, and Bohler’s and Gissane angle of the calcaneus, therefore reaching the anatomical reduction. In this study, the brand of materials used in the ELA group consists of Smith & nephew (29/62), Acumend (10/62), and Double Medical Technology Inc., China (23/62), while in STA group consists of Acumed (31/47) and Carefix, China (16/47). Normally, the option of choosing which type of plates mainly depends on patients’ condition and surgeon’s preferences. However, healthcare system varies in different countries and it can be assumed that there is some bias in choosing internal fixation materials based on patient income and insurance type. When conditions permit, surgeons provide patients with suggestions on therapeutic scheme patients and patients can choose imported or domestic internal fixation materials according to their financial status and wishes. Currently, domestic internal fixation materials have a price advantage and can reach similarly anatomical reduction with low complication rate compared with imported ones. In our study, the patients in the ELA group underwent ORIF with plates and five to eight 3.5-mm screws while the patients in the STA group underwent ORIF with three to four 6.5-mm cannulated screws solely. In the past 20 years, the technique of STA has been introduced in China and there were no comparable implants to match it, and therefore, this technique has not been popularized. Currently, as new generation of implants emerges, more and more surgeons tend to perform minimal invasive incisions and the cost of these implants are comparable to plate system in extensile lateral technique. Whether plate system or screws are applied via two different approaches, there were no definite indications for it. Based on our experience, normally, procedures via ELA do not apply with screws solely and procedures via STA do not apply with large plates. We tend to apply screws for patients with intact calcaneal wall and simple posterior calcaneal articular surface collapse. For those patients with factures of calcaneal anterior process or impingement of fracture fragments, we tend to apply plate systems to reach anatomic reduction. The selection of implants is based on the specific condition of each patient, and we control other parameters including patients’ information and fracture type, and that is the way we justified the cost of the patients.

As for the life of various types of implants, normally, the implants will be removed if situations including pain and infections happen. In our countries, a majority of patients choose to have their implants removed as long as the fracture site reaches clinical healing standard or 24 months after the surgery. However, it may not be possible to track and analyze the cost of removal internal fixations for it may not be performed in the same hospital.

In our study, no severe complications occurred and all the patients at final follow-up observations showed an excellent therapeutic effect after surgery according to direct measurement of clinical outcomes. The results showed that there were no significant differences between the ELA and STA groups among different surgeons on the clinical efficacy indicators. Changes in pre- and postoperative VAS and AOFAS scores are both the most direct measurement and rapid evidence of surgical outcomes. Altogether, the application of surgical incision on ELA or STA among surgeons A, B, and C depends on their own experience and preference. According to the clinical indicators, both incisions have comparative curative effects and equivalent precision ratio on patients with type II/III calcaneus fracture because there were no significant differences. They can alleviate patients’ pain and enable them to take their own responsibilities in the society.

The outcome of CUA was presented with gained QALYs, which were enumerated through multiplying the length of hospital stay by HRQoL weight (i.e., utility score) scaled from − 0.11 to 1.00 [46]. Direct elicitation methods, generic preference-based measures, and condition-specific measures were used to evaluate HRQoL. The EQ-5D-3L, regarded as the primary valuation study derived utility scores, are prevalently applied preference-based measures [47–49]. In this study, CER and ICERs were the outcomes of economic benefits, which could be comprehended that ICERs indicate how much extra health benefits via ELA can bring compared with via STA, and how much additional expense it will cost.

Consequently, we found no statistically significant differences in overall cost per patient and HRQoL between the two groups, but generally, surgery via ELA incurred much costs than via STA. Similarly, the incremental cost-utility ratio also showed there were no cost-utility benefits comparing the two groups.

## Limitation

This study has its own limitations. Firstly, the chief limitation of study is that it is a retrospective cost-utility analysis and randomized clinical trials (RCTs) are insufficient. Therefore, there are inherent defect existing in the study. Secondly, all the data are collected from a sing center, which leads to the fact that the sample size is not adequate and it may result in single center analysis bias. Thirdly, indirect costs including rehabilitation, home care, and further consultation have not been collected as patients may go back to local hospital and produce fees that could not be tracked. Fourthly, only patients classified as Sanders type II/III were focused solely on in the study and future RCTs combined with CUA could emphasize on other types of calcaneus fractures such as Sanders I/IV, malunion, and nonunion. Lastly, there did exist some level of recall bias in patients’ accomplished questionnaires and limitation of all CUA in lacking of uniform methodology to track the preoperative outcome.

## Conclusion

Both ELA and STA techniques are effective surgical procedures for the patients with calcaneus fracture. Moreover, STA seems to be more reasonable for its merits including less postoperative pain, fewer expenses of analgesia, and internal fixation materials.

## Data Availability

The datasets used and/or analyzed during the current study are available from the corresponding author upon reasonable request.

## Abbreviations

ELA: Extensile lateral approach
STA: Sinus tarsi approach
DIACFs: Displaced intra-articular calcaneus fractures
CUA: Cost-utility analysis
ORIF: Open reduction and internal fixation
ICERs: Incremental cost-effectiveness ratio
HRQoL: Health-related quality of life
VAS: Visual analogue scale
EQ-5D: EuroQol five-dimensional
AOFAS: American orthopaedic foot and ankle society
QALY: Quality-adjusted life-year
